# Data-Centric AI for EEG-Based Emotion Recognition: Noise Filtering and Augmentation Strategies

**DOI:** 10.3390/bioengineering12111264

**Published:** 2025-11-18

**Authors:** Nadieh Moghadam, Rana Hegazy

**Affiliations:** 1 Department of Electrical Engineering, University of San Diego, San Diego, CA 92110, USA; rhegazy@sandiego.edu; 2 Electronics and Electrical Communications Engineering Department, Cairo University, Giza 12613, Egypt

**Keywords:** artificial intelligence, data-centric AI, emotion prediction, medical signal

## Abstract

Research in the biomedical field often faces challenges due to the scarcity and high cost of data, which significantly limit the development and application of machine learning models. This paper introduces a data-centric AI framework for EEG-based emotion recognition that emphasizes improving data quality rather than model complexity. Instead of proposing a deep architecture, we demonstrate how participant-guided noise filtering combined with systematic data augmentation can substantially enhance system performance across multiple classification settings: binary (high vs. low arousal), four-quadrant emotions, and seven discrete emotions. Using the SEED-VII dataset, we show that these strategies consistently improve accuracy and F1 scores, achieving competitive or superior performance compared to more sophisticated published models. The findings highlight a practical and reproducible pathway for advancing biomedical AI systems, showing that prioritizing data quality over architectural novelty yields robust and generalizable improvements in emotion recognition.

## 1. Introduction

Data-Centric Artificial Intelligence (DCAI) has emerged as a crucial approach in improving the performance of AI systems. Unlike the traditional emphasis on designing increasingly complex AI models, DCAI focuses on optimizing the quality of the data used to train these models. Recent studies have shown that cleaner, more consistent and less noisy datasets can yield performance improvements equivalent to, or even surpassing, those achieved by doubling or tripling the size of the dataset [1]. This paradigm shift underscores the transition from a “big data” mindset to a “good data” philosophy.

The foundation of DCAI lies in improved data preparation and management. Ensuring consistent labeling across output data is a crucial factor. For instance, labeling inconsistencies can introduce noise and degrade model performance even when sophisticated models are employed. Moreover, addressing important edge cases in the input data ensures that the model learns robustly across a broader spectrum of scenarios. This can significantly reduce biases and blind spots in predictions, particularly in domains like healthcare and finance, where edge cases often carry high stakes. Appropriately sizing datasets is another key tenet of DCAI. Instead of arbitrarily increasing dataset size, the focus is on selecting data that is representative of the problem space while avoiding redundant or irrelevant information. Tools such as active learning, which identify the most informative data points for labeling, have proven effective in achieving this balance [2]. Furthermore, DCAI emphasizes the importance of monitoring and addressing data drift, a phenomenon in which the statistical properties of input data change over time, using techniques like continuous monitoring and data versioning to adapt to these shifts [3].

Data augmentation is a core component of DCAI, especially in biomedical and healthcare domains where both data scarcity and noise are common. Recent reviews and applications have shown its role in improving foundation models, digital twins, and clinical decision-support systems [4,5,6,7,8,9,10,11]. In related research, multimodal and wearable-sensor studies demonstrate that integrating physiological and behavioral signals enhances emotion and health prediction [12,13,14,15]. For EEG applications specifically, George et al. [16] showed that task-specific data augmentation can markedly improve motor-imagery decoding, emphasizing the value of generating high-fidelity synthetic neural data.

In the context of EEG-based emotion recognition, DCAI provides a structured approach to tackle two major challenges: limited high-quality labeled data and substantial signal noise. EEG recordings are vulnerable to electrode displacement, motion artifacts, and subject-specific variability, all of which can distort the relationship between signals and emotional labels. Rather than relying on larger or deeper models, a DCAI strategy seeks to enhance the reliability of training data through systematic noise filtering, cleaning, and validation of labels. Recent work has also emphasized the importance of EEG data quality itself. For example, Kalita et al. [17] proposed an LSTM–GAN framework for automated EEG artifact removal and demonstrated that deep-learning–based denoising substantially improves signal quality. Similarly, Hill et al. [18] introduced the RELAX-Jr pipeline, which systematically compared multi-stage automated EEG cleaning methods using metrics such as signal-to-error and artifact-to-residue ratios, further underscoring that optimizing EEG data quality is a critical and ongoing focus in current research.

The SEED dataset is a collection of EEG datasets corresponding to specific emotions. It has several versions. Many studies have been conducted on the older versions of the dataset (SEED [19], SEED-IV [20], SEED-V [21]). In [22], emotion recognition is performed using a model based on Long Short-Term Memory neural networks. The model can distinguish non-linear relationships among EEG signals from different electrodes and aims to narrow the distribution gap between training and test sets. On the other hand, ref. [23] proposed a graph-based multi-task self-supervised learning model for emotion recognition. The work is divided into spatial and frequency jigsaw tasks, in addition to contrastive learning tasks, to learn more general representations. The two emotion recognition models, deep canonical correlation analysis and bimodal deep autoencoder, are compared in [21]. The former demonstrated superior performance across different datasets and showed robustness to noise. In [24], a regularized graph neural network is developed for EEG-based emotion recognition. The inter-channel relationships in EEG signals are modeled using adjacency matrices, and two regularizers are proposed to address cross-subject EEG variations.

To address data scarcity, numerous works have applied augmentation to EEG-based emotion recognition. Most targeted earlier SEED versions [25,26] or other EEG dataset [27,28]. Tian et al. [25] employed a dual-encoder VAE-GAN to synthesize spatiotemporal EEG features, improving cross-subject accuracy by about 5%. Luo et al. [26] found that generating synthetic samples roughly one-tenth the size of the original dataset produced optimal gains. Krell et al. [27] simulated electrode-cap shifts by rotating sensor coordinates, creating plausible new trials. Lotte et al. [28] generated artificial EEG segments via time-, frequency-, and time–frequency-domain distortions, reducing calibration effort in brain–computer interfaces.

SEED-VII [29], the dataset used in this study, is a recent addition to the SEED family. It was originally introduced alongside the Multimodal Adaptive Emotion Transformer (MAET) model [30], which combined EEG and eye-movement data for multimodal emotion recognition. Few studies have utilized it to date. In [31], Fourier-inspired techniques are introduced to separate periodic and aperiodic components of EEG signals for improved feature extraction. In [32], a ChannelMix-based transformer and convolutional multi-view feature fusion network is proposed to enhance cross-subject emotion recognition by capturing richer spatiotemporal representations.

In this work, we adopt a *data-centric* perspective for EEG-based emotion recognition using SEED-VII. We focus exclusively on EEG data to predict (i) high/low arousal states, (ii) four quadrants of the Russell two dimensional emotion model, and (iii) seven discrete emotions. The novelty of this study lies in filtering noisy samples based on participants’ self-reported emotion scores and coupling this cleaning process with label-preserving data augmentation strategies. Unlike prior work that applied augmentation in isolation [25,26], our approach integrates participant-driven noise filtering and augmentation within a unified DCAI pipeline. All experiments employ a consistent five-fold cross-validation procedure to ensure statistical reliability and generalizability. Building on our earlier data-centric work on medical datasets [33], this study extends the methodology to EEG emotion recognition.

**Research Objective.** The objective of this study is to evaluate whether a DCAI pipeline that combines participant-score-guided noise filtering and label-preserving data augmentations can significantly improve EEG-based emotion recognition on SEED-VII across (i) binary arousal, (ii) four-quadrant, and (iii) seven-class classification tasks under consistent five-fold cross-validation.

**Contributions.**

- We introduce a DCAI preprocessing framework for EEG-based emotion recognition that removes low-confidence samples using participants’ self-reported emotion scores and integrates multiple data augmentation strategies including averaging-based, Gaussian, and time-based augmentations to mitigate data scarcity.
- We evaluate the framework across three classification tasks using consistent five-fold cross-validation, isolating the impact of data quality and augmentation from model complexity.
- We show that the proposed approach remains robust under Principal Component Analysis (PCA)-based dimensionality reduction, confirming that the observed performance gains stem from improved data quality rather than feature count.

The remainder of this paper is organized as follows. Section 2 introduces the SEED-VII dataset and the emotion categorization framework, followed by a description of the deep-learning architectures in Section 3. Section 4 presents the experimental results and system performance analysis. Section 5 provides a detailed discussion of the findings, highlighting their theoretical implications, relation to prior work, and limitations. Finally, Section 6 concludes the study and outlines future directions for extending the proposed framework to other datasets and modalities.

## 2. Dataset and Emotion Categorization Framework

### 2.1. Dataset

The dataset utilized in this study, as described in [29], was designed to facilitate the prediction of emotions based on physiological signals, specifically EEG data. It captures participants’ emotional responses to video stimuli, providing a well-structured and systematic dataset for emotion prediction tasks. The dataset includes annotations for six distinct emotions: happy, sad, disgust, fear, surprise, and anger, along with a neutral state. This section outlines the structure of the dataset, the experimental procedure, and the features derived from EEG signals.

The dataset comprises 12 video clips for each of the six emotions, while the neutral state is represented by eight clips. Each video clip lasts between two to five minutes, providing sufficient duration to evoke and measure emotional responses. Participants viewed these clips in five separate sessions, with each session spaced at least 24 h apart to minimize fatigue and ensure the validity of emotional responses. During each session, 20 video clips corresponding to one of the six emotions were displayed. After viewing each clip, participants provided feedback by rating the intensity of the emotion elicited by the clip. The ratings ranged from 0 to 1, where 0 indicated no emotional response and 1 represented a strong emotional response. A total of 20 participants contributed to this study, ensuring a robust and diverse dataset.

The EEG data used in this study underwent preprocessing to enhance its utility for emotion prediction. Differential entropy was applied to the EEG signals, transforming them into features that capture the statistical characteristics of the brain’s electrical activity. Since the duration of the video clips varies, the resulting EEG recordings differ in the number of time samples. To ensure a consistent input size for the model, each sample was averaged over time. The EEG data comprises five frequency bands: delta, theta, alpha, beta, and gamma, each associated with distinct brainwave activity. Each frequency band includes signals from 62 EEG channels, yielding a total of 310 features (5 bands × 62 channels) per emotion instance. These features are used as the input to the deep learning model, providing a comprehensive representation of the participants’ neurological responses to emotional stimuli.

This carefully designed and processed dataset forms the foundation of our study, enabling the application of advanced deep learning techniques to predict emotional states with high accuracy.

### 2.2. Emotion Categorization

To classify emotions based on their intensity and valence, we adopt the Russell 2D model [34], a widely recognized framework for emotion representation. In this model, emotions are mapped within a two-dimensional space defined by *Valence* (positive vs. negative) and *Arousal* (high vs. low).

**Figure 1:**
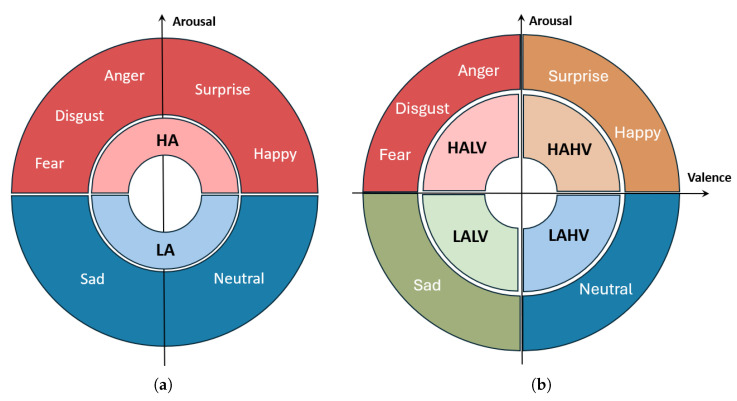
Visualization of emotion classification: (**a**) High Arousal (HA) and Low Arousal (LA) emotions. (**b**) Mapping emotions across the four quadrants.

Building on this model, we first simplify the classification into two main categories based on arousal level: *High Arousal*, (*HA*) and *Low Arousal*, (*LA*). Specifically, the emotions *happy*, *disgust*, *fear*, *surprise*, and *anger* are categorized as HA emotions, while *sad* and *neutral* are classified as LA emotions. In the Russell 2D model, arousal is defined as the level of physiological activation. We group *neutral* and *sad* as low arousal (LA) since both typically elicit low physiological activation and subdued EEG patterns. While neutral could also be interpreted as neither high nor low arousal, prior studies [19,22] have categorized it as LA for practical classification. This binary classification provides a focused approach to predicting emotional arousal levels, aiding the development of a more efficient emotion prediction system. Figure 1a illustrates this classification, visually distinguishing between high arousal and low arousal emotions.

Expanding beyond binary classification, we also investigate the effect of predicting four-quadrant emotion categories:

- High Arousal High Valence (HAHV): Happy, surprise.
- High Arousal Low Valence (HALV): Disgust, anger, fear.
- Low Arousal Low Valence (LALV): Sad.
- Low Arousal High Valence (LAHV): Neutral.

By predicting four distinct emotional states instead of two, the model’s task becomes more complex, as it must differentiate between subtle variations in valence alongside arousal levels. This finer classification leads to less available data per category, which can impact performance, a challenge we address in the following sections. Figure 1b illustrates this expanded categorization, mapping emotions across the four quadrants of the Russell 2D model.

The dataset’s emotion distribution across quadrants is uneven:

- 45% of data falls within the HALV quadrant,
- 30% in HAHV,
- 15% in LALV, and 
- 10% in LAHV.

Notably, neutral emotion (LAHV) has only 8 video clips, whereas each of the other emotions is represented by 12 clips. This imbalance introduces additional challenges in training the model effectively across all quadrants. The impact of this categorization on model performance is discussed in Section 4.

Lastly, all the EEG data corresponding to the different emotions are separated and each emotion is predicted separately from the other emotions.

## 3. Deep Learning Model

Our proposed deep learning model processes EEG-derived features to predict emotional states. The architecture consists of a 1D convolutional layer, pooling, dropout layers for regularization, and dense layers for classification.

- **Convolutional Layer (Conv1D):** The first layer applies 64 filters with a kernel size of 3 and the ReLU activation function to extract temporal patterns from the input features.
- **Pooling Layer:** A MaxPooling1D layer with a pool size of 2 reduces dimensionality and emphasizes the most salient features.
- **Dropout:** To mitigate overfitting, dropout layers with rates between 0.25 and 0.5 were used at different stages of the network, depending on the architecture variant.
- **Flatten and Dense Layers:** After feature extraction, the output is flattened and passed to fully connected dense layers. The final output layer uses a sigmoid activation for binary classification (two halves) and softmax for multi-class settings (four quadrants and seven emotions).

The models were trained for 50 epochs with a learning rate of 0.0001 using the Adam optimizer.

### 3.1. Performance Metrics

Model performance was evaluated using two metrics: *accuracy* and *F1 score*. Accuracy measures the proportion of correctly classified samples, while the F1 score balances precision and recall, providing a more reliable assessment under class imbalance.

The following terms are used to calculate the F1 score:

- True positive (TP): The model correctly predicts an emotion.
- False negative (FN): The model fails to predict an emotion when it is the correct output.
- False Positive (FP): The model predicts an emotion incorrectly.

Using these metrics, precision and recall are defined as:

(1) $$\begin{array}{c} Precision=\frac{TP}{TP+FP} \end{array}$$

(2) $$\begin{array}{c} Recall=\frac{TP}{TP+FN} \end{array}$$

The F1 score is then calculated as

(3) $$\begin{array}{c} F1\;\mathrm{score}=\frac{2\cdot \mathrm{Precision}\cdot \mathrm{Recall}}{\mathrm{Precision}+\mathrm{Recall}} \end{array}$$

The F1 score is particularly valuable for unbalanced datasets, where some classes may have significantly fewer samples than others.

### 3.2. Ablation Studies

To identify the best-performing model, we conducted ablation studies across multiple architectures. Evaluation was performed using five-fold cross-validation, with performance reported as the mean across folds. The goals of the ablation studies were: (1) to determine the most effective model architecture, and (2) to evaluate the impact of data augmentation techniques on accuracy and robustness.

Table 1 summarizes the performance of different deep learning models tested with varying parameters across the three emotion categorizations: two halves, four quadrants, and seven emotions.

Each model was evaluated based on its accuracy and F1 score. Among the models tested, Model 1 achieved the highest accuracy and F1 score for the two-half case, while Model 2 performance surpasses the other two models in the case of four quadrants and seven emotions. These results demonstrate that as the number of predicted classes increase, given the same total number of data points, more layers are needed, and the accurate prediction of the classes decreases.

With the best model identified for each emotion categorization, we proceeded to investigate the effect of data cleaning and augmentation on system performance as detailed in the next section.

## 4. System Performance and Results

To evaluate the effectiveness of data centric strategies, we conducted experiments measuring the impact of data cleaning (noise filtering) and data augmentation on model performance. All evaluations were performed in a five-fold cross-validation framework: the full dataset was randomly divided into five folds of equal size, four of which were used for training (and validation) and the remaining one for testing, rotating so that each fold served as the test set once.

**Figure 2:**
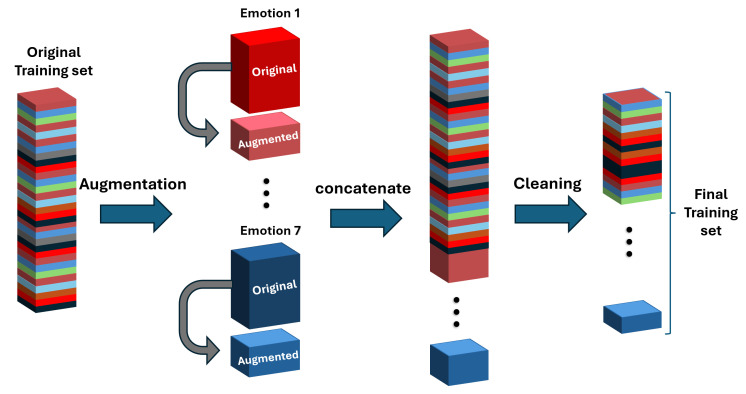
Illustration of the proposed Data-Centric AI pipeline.

Figure 2 provides an overview of our DCAI pipeline applied to the training process. After splitting the data into training and test folds, we first perform data augmentation on the training set and then apply noise filtering (removing low-confidence samples). It is important to note that augmentations are applied *only* to the training data; the test data is never augmented, which prevents any information from the test set from leaking into training. Both the augmentation and cleaning steps are label-preserving, meaning they do not alter the target emotion labels of the data.

### 4.1. Data Augmentation and Noise Filtering

**Terminology.** In this work, *Data-Centric AI* (*DCAI*) refers to optimizing data quality rather than increasing model complexity. Within this framework, *data cleaning* and *noise filtering* that are used interchangeably, denote the removal of unreliable samples based on participant self-reports, whereas *data augmentation* refers to label-preserving synthesis techniques (averaging, Gaussian, and time-based).

With these definitions established, we next describe how these processes were applied in practice.

To address the trade-off between data cleanliness and dataset size, we employ three label-preserving augmentation strategies: For data augmentation, we implemented three different strategies:

1. **Averaging-based augmentation:** Synthetic samples are created by element-wise averaging of existing feature vectors from the same emotion class. We experimented with different group sizes averaging pairs of samples and groups of three to five consecutive samples. Among these, the method that averages five same-class entries consistently achieved the best performance. Therefore, throughout this study, all references to “averaging-based augmentation” correspond to this configuration.
2. **Gaussian augmentation:** Zero-mean Gaussian noise (nominal SNR = 15 dB) was added to the 310-dimensional DE feature vectors [35]. The noise variance was scaled to each sample’s signal power and randomly modulated within 0.5–1.5× to ensure variability. The augmented samples were concatenated with the originals, doubling the training set size. This technique improves model robustness by simulating minor variations in EEG features that could occur due to sensor noise or individual differences, without changing the underlying emotion label.
3. **Time-domain augmentation:** We leveraged the fact that multiple participants watched the same video clips to create new synthetic samples. For two different participants who viewed the same emotional clip, their EEG time-series signals were averaged sample-wise to generate a new trial, ensuring frame-level temporal alignment before differential-entropy feature extraction. The label for each augmented trial was set as the mean of the two participants’ self-reported emotion scores, and augmented data involving test participants were excluded to prevent data leakage.

Averaging-based, Gaussian, and time-domain augmentations were evaluated in separate experiments and were never combined within the same training pipeline. Each produced an independent set of results.

To prevent data leakage, all cross-validation splits were performed at the subject level. Data from test participants were completely excluded from the training process, including all augmentation operations, ensuring that no EEG samples or synthetic data derived from them appeared in the training folds. All augmentations were applied only to the training folds, while the test fold was used exclusively for evaluation.

This setup effectively prevents temporal or subject-level information leakage; however, a further consideration arises due to the shared video stimuli across participants. Although each EEG trial is represented by a single differential-entropy (DE) feature vector that prevents temporal overlap, the current five-fold cross-validation may include EEG responses to the same video stimulus from different participants in both training and testing folds. This protocol follows the standard SEED-VII evaluation practice reported by Jiang et al. [29]; however, future work will incorporate clip-disjoint and subject-independent splits to ensure complete separation between training and testing data.

Participants’ self-reported emotion scores are used to validate the predefined emotion labels assigned to each video. Because participants may not always experience the intended emotion with the same intensity, these self-reports serve as indicators of label reliability rather than alternative labels. Evidence from recent multimodal affective studies such as the Emognition dataset [36] and the EEG-based emotion dataset [37] shows that incorporating self-reported affect alongside stimulus labels improves the emotional validity of datasets and the robustness of the models. Following these insights, in this work, the participants’ emotion intensity scores in SEED-VII are leveraged to guide a participant-informed noise filtering step.

After data augmentation, we apply *noise filtering* using the participants’ self-reported emotion intensity scores as a proxy for label confidence. Each SEED-VII sample (video clip viewing) includes such a score, reflecting the participant’s perceived emotional intensity. Samples with very low ratings (close to 0) are considered less reliable, as the corresponding clips may not have successfully triggered the intended emotion or the participant may have been disengaged. We introduce a threshold parameter, termed the *cleaning ratio*, to remove these low-confidence data points. For example, a threshold of 0.2 eliminates all samples where the participant’s reported emotion intensity is ≤0.2, whereas a threshold of 0.95 retains only the strongest emotional responses. We systematically vary this threshold to examine the trade-off between data quantity and quality.

We report the model’s accuracy and F1 score under different cleaning thresholds and augmentation strategies in Table 2, Table 3 and Table 4. In these tables, the baseline condition corresponds to the first entry in the *Original* column (cleaning ratio = 0), representing the model trained without data cleaning or augmentation. As progressively more aggressive cleaning is applied (moving down each column of the tables), both accuracy and F1 generally improve despite the reduction in training set size. This trend supports the data-centric viewpoint that improving data quality, even at the cost of quantity, can enhance model performance. For example, in the two-category arousal classification (Table 2), using only the top 12.5% of the data (threshold 0.95, which leaves about 200 of the most confident samples) yields better performance than using 100% of the data with all the noisier samples included.

It is worth emphasizing that this noise filtering strategy crucially relies on having the participants’ feedback scores as a measure of label confidence. In many real-world datasets, such additional information may not be available, which would make identifying and removing low-confidence samples more difficult. Our results highlight the value of collecting auxiliary information (like self-reported emotion intensity) during data acquisition. By evaluating the reliability of each label, one can markedly improve the overall quality of the dataset and, as a result, the performance and generalizability of the trained model.

As a consequence of applying these thresholds, higher cleaning ratios reduce the number of low-confidence samples and alter class balance. Table 5 summarizes the class distributions after applying each cleaning threshold, showing that higher thresholds substantially reduce the dataset size and affect class proportions.

### 4.2. Statistical and Dimensionality Analyses

To evaluate the robustness of the proposed DCAI preprocessing framework to feature dimensionality, we additionally applied Principal Component Analysis (PCA) as a postprocessing step to the extracted feature vectors. We explored multiple PCA configurations, including a fixed number of components and different variance-retention ratios. Among these, the PCA with a 0.99 variance-retention threshold achieved the best overall performance, reducing the 310 differential-entropy features to approximately 180–40 components across different cleaning ratios while maintaining or slightly improving classification accuracy. This variation in component count arises because removing low-confidence samples changes the data covariance structure; cleaner datasets exhibit more compact variance distributions, requiring fewer components to retain the same 99% of total variance. For each cross-validation fold, PCA was fit exclusively on the training data and subsequently applied to the corresponding test data using the same fitted transformation, thereby preventing any data leakage. The number of retained principal components was fixed per cleaning ratio but varied slightly across folds due to minor covariance differences, all while maintaining the fixed 99% variance-retention threshold. All other preprocessing, cleaning, and augmentation steps were kept identical across both the PCA and non-PCA configurations.

Across all tested PCA configurations, performance metrics remained stable, with accuracy and F1 variations within the standard deviation range observed in the non-PCA setup. This confirms that the observed improvements originate from the DCAI preprocessing, particularly noise cleaning and augmentation rather than from feature dimensionality reduction. In other words, applying PCA neither degraded nor significantly enhanced model performance, highlighting the intrinsic robustness of the cleaned data representation. This trend is also evident in Table 2, Table 3 and Table 4.

Confidence intervals were computed across the cross-validation folds using the standard normal approximation:

(4) $$\begin{array}{c} {\mathrm{CI}}_{95}=z_{0.975}\times \frac{\mathrm{SD}}{\sqrt{n}} \end{array}$$

where $z_{0.975}$ is the critical value of the standard normal distribution corresponding to 95% confidence (1.96 for a two-tailed interval), SD denotes the standard deviation across folds, and *n* is the number of folds (five in this study).

**Figure 3:**
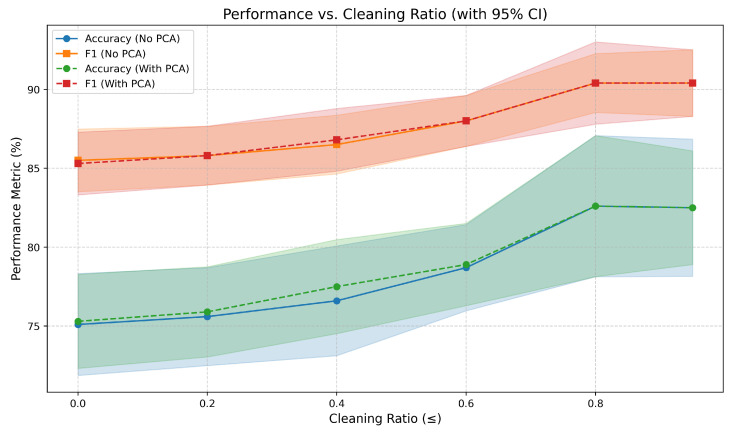
Accuracy and F1 (mean ± 95% CI) as a function of cleaning ratio for both PCA and non- PCA configurations.

Figure 3 shows the model’s accuracy and F1 (mean ± 95% CI) versus cleaning ratio for both PCA and non-PCA configurations. As the cleaning ratio increases, both accuracy and F1 improve steadily while remaining within narrow uncertainty bounds, confirming that the model’s generalization performance is stable despite the reduced data volume. These results illustrate the expected trade-off between data quantity and quality, consistent with the data-centric learning perspective emphasized in this work.

Beyond evaluating the effect of dimensionality reduction, we also assessed statistical consistency across folds to ensure reliable cross-validation performance.

Table 6 summarizes the detailed statistical results across augmentation strategies (Original, Averaging-based, and Time-domain) with and without PCA. Each entry reports the mean and standard deviation of both accuracy and F1 score over the five cross-validation folds. The standard deviations remain within a narrow 1.3–3.6% range across all configurations, indicating consistent generalization and stable model behavior. Paired t-tests conducted across folds confirmed that the majority of improvements were statistically significant (p<0.05).

Next, we will study performance of the system, when noise reduction and data augmentation are used, in case of two-halves, four-quadrants, and seven-emotion classifications.

The complete data processing and evaluation procedure is summarized in Algorithm 1. This pseudocode outlines the Data-Centric AI workflow adopted in this study, detailing the sequential application of augmentation, participant-guided noise filtering, and model training within a five-fold cross-validation framework to ensure methodological clarity and reproducibility.
**Algorithm 1** Data-Centric EEG Emotion Recognition Pipeline.
 **Input:** SEED-VII EEG dataset with participant self-reported emotion scores **Output:** Mean ± SD of accuracy and F1-score across five folds1:Split dataset into five folds $\left(F_{1},F_{2},F_{3},F_{4},F_{5}\right)$2:**for** each fold $F_{i}$ as test set **do**3:    Use remaining four folds as training set4:    Apply **data augmentation** on training set:
Averaging-based augmentationGaussian noise augmentationTime-domain augmentation5:    Apply **participant-guided noise filtering**:
Remove samples with emotion intensity ≤ threshold (e.g., 0.2–0.95)6:    **Optional PCA:** fit PCA on *training* data;7:    Train CNN model for 50 epochs
Optimizer: AdamLearning rate: 0.00018:    Evaluate model on untouched test fold $F_{i}$9:**end for**10:Compute mean ± SD of Accuracy and F1-score across folds

### 4.3. Impact of Predicting Two-Halves Emotions

Table 2 illustrates the effect of data cleaning and augmentation on predicting the two-halves emotion categories, high-arousal and low-arousal. The baseline case is the top-left entry of the table, corresponding to the Original dataset without any data cleaning, which achieves an accuracy of 74.2%. As the cleaning ratio increases, the system’s performance improves consistently across all datasets (Original, Average Augmentation, and Time-Augmentation), both with and without PCA.

For the Original dataset without PCA, accuracy rises from 74.2% to 82.5% at 0.95 cleaning ratio, representing overall gain of +8.3 percentage points. The Average Augmentation configuration follows a similar trend and accuracy improvement. The Time-Augmentation method increases from 72.0% to 82.2% at ratio 0.95, an enhancement of approximately +10.2 percentage points. The Gaussian-based augmentation achieves accuracy values comparable to or slightly higher than the averaging-based variant across most cleaning thresholds, confirming that minor, label-preserving perturbations in the feature domain can effectively enhance generalization without altering class consistency.

Applying PCA (0.99 variance-retention ratio) produces nearly identical accuracies across all cleaning ratios, confirming that dimensionality reduction has negligible impact on performance. Overall, the results indicate that data cleaning is the main driver of improved accuracy, while data augmentation offers only minor gains. The model’s stability under PCA further supports the DCAI principle that high-quality preprocessing, rather than feature quantity, determines robust and generalizable performance.

### 4.4. Impact of Multi-Class Emotion Prediction (Four-Quadrant and Seven-Emotion)

To further evaluate the scalability of the proposed DCAI framework, we extended the analysis from the binary two-halves emotion classification to more complex multi-class settings: the four-quadrant and seven-emotion tasks. Both tasks follow a similar trend to the two-halves case, with performance improving consistently as the cleaning ratio increases.

Table 4 also includes the MAET model’s EEG-only baseline from [30], which achieved comparable accuracy under the seven-class configuration. MAET was reproduced following the official configuration of [30], using only the EEG modality of the SEED-VII dataset. All model parameters, optimizer settings, and training procedures were kept identical to the reference implementation.

After applying our cleaning pipeline, MAET’s performance rose to 45.9%, matching the results of our simpler model trained on cleaned and augmented data. This demonstrates that high-quality data can enable lightweight models to rival complex architectures and that data-centric and model-centric improvements are complementary.

Across both experiments, data cleaning is the primary driver of accuracy gains, producing improvements of approximately 15–17 percentage points from the baseline to the optimal level (0.95). Data augmentation offers modest additional improvements of less than 2 percentage points, while applying PCA with a 0.99 variance-retention ratio yields nearly identical results to the non-PCA configuration.

Although overall accuracy decreases as the number of emotion classes increases from approximately 82% for two-halves to 65% for four-quadrant and 45% for seven-emotion, the performance trends remain consistent. These findings confirm that the DCAI framework generalizes effectively across increasing task complexity, with model robustness driven primarily by the quality of the cleaned and augmented data rather than the dimensionality of the feature space.

**Figure 4:**
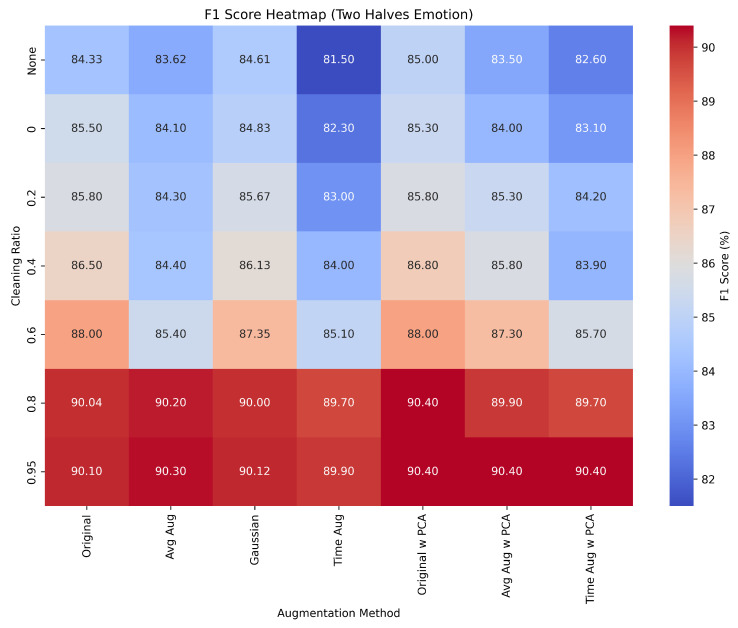
F1 Score heatmap for Two Halves Emotion under various data cleaning and augmentation.

Figure 4 presents the F1-score heatmap for the *Two-Halves* emotion classification task under varying data-cleaning thresholds and augmentation strategies. The heatmap provides a concise summary of the comparative performance across augmentation methods and PCA. A consistent upward trend in F1-score is observed as low-confidence samples are removed, confirming that data cleaning improves generalization. Among the augmentation approaches, *Gaussian* augmentation yields slightly higher F1-scores across most thresholds, particularly between 0.4 and 0.8 cleaning ratio. Models incorporating PCA achieve comparable or marginally improved scores, indicating that dimensionality reduction reduces feature redundancy and stabilizes performance.

Overall, this visualization captures the general trend across all classification settings, demonstrating that targeted data cleaning combined with moderate augmentation substantially improves EEG-based emotion recognition performance. Similar patterns are also observed in the *Four-Quadrant* and *Seven-Emotion* scenarios, confirming that these gains stem primarily from improved data quality rather than model complexity.

Table 7 summarizes the highest F1 scores achieved in the three emotion classification setups (Two Halves, Four Quadrants, and Seven Emotions), along with their respective data-cleaning thresholds. Across all configurations, the inclusion of data cleaning and augmentation leads to consistent performance improvements over the unfiltered baseline.

Across all setups, the Two-Halves configuration with any augmentation method and PCA reaches the highest F1 score of 90.4% at cleaning ratios between 0.8 and 0.95, marking an improvement of approximately +7.2% compared to the unfiltered baseline (84.33%). For the Four-Quadrant classification, Average Augmentation yields the top F1 score of 44.1% at a cleaning ratio of 0.2, corresponding to an improvement of +29.7% over the baseline (34%). In the Seven-Emotion setup, the best overall F1 score of 38.6% is also achieved by the Average Augmentation at a cleaning ratio of 0.95, reflecting a relative increase of about +38.8%.

## 5. Discussion

The quantitative evaluation shows that participant-guided noise filtering and targeted augmentation lead to measurable performance gains across all labeling schemes. These findings provide empirical support for the Data-Centric AI (DCAI) principle that optimizing data and label reliability enhances EEG-based emotion recognition without added model complexity.

This approach aligns closely with data-oriented engineering (DOE) principles, emphasizing the optimization of input data as the foundation of system performance. Consistent results across five-fold cross-validation confirm the reproducibility and robustness of these improvements. Moreover, the negligible impact of dimensionality reduction through Principal Component Analysis (PCA) underscores that performance gains arise from data reliability rather than feature quantity or model depth. Collectively, these insights validate that the DCAI framework enhances generalization through cleaner, more representative input signals rather than through architectural modifications.

### 5.1. Relation to Prior Work and Practical Implications

Compared with prior EEG emotion recognition studies such as Zheng and Lu [19], Li et al. [23], and Luo et al. [26], which primarily emphasize model innovation or generative augmentation, this work provides a complementary perspective that enhances data reliability as a means of improving model performance.

Kalita et al. [17] and Hill et al. [18] further illustrate the recent shift toward data-centric EEG research. Both studies focus on improving EEG quality through automated or deep-learning–based denoising. While these works demonstrate the measurable impact on signal fidelity, they do not examine how such preprocessing influences affective classification tasks. The present study extends this perspective by quantifying the effect of targeted cleaning and augmentation on emotion-recognition accuracy, thereby linking signal-quality optimization to model-level outcomes within a unified DCAI framework.

Although some augmentation strategies did not yield consistent performance gains, this finding is itself informative. The results suggest that augmentation benefits depend on the baseline data variability and the noise characteristics of the dataset. For example, when the dataset is already clean (e.g., after high-threshold filtering), additional synthetic variability may introduce redundancy rather than diversity. This emphasizes the need for task-aware and imbalance-sensitive augmentation methods in future work. Interestingly, the impact of augmentation varied with task complexity. As shown in Table 2, Table 3 and Table 4, augmentation produced minimal or even negative effects in the two-halves classification but yielded clearer gains in the four-quadrant and seven-emotion settings. This pattern reflects the interplay between task difficulty and sample diversity. In the binary arousal task, each class already aggregates multiple emotional states, providing substantial intra-class variability and clear inter-class separation. Under such conditions, additional synthetic samples may introduce redundant or noisy variance, slightly degrading performance. In contrast, in the four- and seven-class problems, where the data are more fragmented and sparse, augmentation helps by generating synthetic samples that increase intra-class diversity and improve model generalization. These results indicate that the benefit of augmentation grows with class granularity and data sparsity, supporting the view that augmentation is most effective when the baseline dataset lacks diversity rather than when it is already clean and well represented.

From a practical standpoint, these findings have important implications for real-world EEG applications. In contexts such as wearable neurotechnology, affect-aware human–computer interfaces, and mental health monitoring, high-quality data acquisition is costly and often limited. The proposed DCAI framework, with augmentation and robust preprocessing, provides a computationally efficient solution that maintains strong generalization even under constrained data conditions. Its stability under PCA-based dimensionality reduction and time-domain augmentation further suggests suitability for embedded systems or low-channel EEG configurations, making it highly adaptable for practical deployment.

The participant-guided filtering strategy introduced in this work offers a practical and generalizable means of improving data reliability in EEG emotion recognition. Unlike previous studies that often assume ground-truth labels are fully reliable, our approach integrates participants’ self-reported emotion scores into the preprocessing pipeline to identify and remove ambiguous or low-confidence samples. This aligns with a broader trend in biosignal research toward human-in-the-loop data curation. Recent EEG and multimodal affective studies on datasets such as SEED-IV have demonstrated that incorporating participants’ feedback during preprocessing improves the emotional validity of datasets and the robustness models [36,37]. By treating these subjective feedback signals as informative quality indicators, future emotion-recognition systems can combine algorithmic and participant-informed criteria to enhance data-centric preprocessing pipelines.

### 5.2. Limitations and Future Directions

While the DCAI framework demonstrates clear performance advantages, several limitations should be acknowledged. First, aggressive noise filtering inevitably reduces the dataset size and may increase fold-to-fold variability, potentially limiting generalization to unseen participants or external datasets. In addition, higher cleaning thresholds can disproportionately remove low-intensity samples, introducing class imbalance. Future work could address this issue through imbalance-aware or generative augmentation techniques that restore representational balance across emotion categories while preserving the benefits of data cleaning. Second, the reliance on self-reported emotion scores introduces subjective bias, as such ratings may vary across individuals. Nevertheless, these self-reports were used solely to assess label confidence and guide data cleaning rather than to redefine emotion categories. This approach provides a practical proxy for emotional reliability, consistent with recent multimodal affective studies [36,37].

The generalizability of the framework can be further tested through cross-dataset studies involving datasets with fewer electrodes or alternative spatial configurations, such as DEAP (32 channels) [38] and DREAMER (14 channels) [39]. Since the approach operates on per-channel differential entropy features, the same preprocessing pipeline can be retrained and adapted to new electrode layouts, supporting domain transfer and scalability across heterogeneous EEG recording environments.

## 6. Conclusions

This work validates a Data-Centric AI framework for EEG-based emotion recognition: participant-guided filtering and lightweight augmentation consistently improve performance (accuracy/F1) across binary, four-quadrant, and seven-class settings. The gains persist with PCA, indicating that the benefit comes from higher data/label quality rather than feature count or model depth.

From an application perspective, the proposed framework offers a practical and scalable foundation for affective computing in mental health monitoring, adaptive learning, and emotion-aware interfaces, and it remains suitable for hardware-constrained wearables.

Future research will focus on extending the proposed framework through cross-dataset validation (e.g., DEAP, DREAMER), the development of adaptive noise-thresholding techniques, and the incorporation of multimodal biosignals to enhance generalization and enable real-time emotion tracking in both clinical and wearable applications. In addition, we will investigate advanced augmentation strategies, including GAN- and VAE-based generative methods, to further increase data diversity and complement the Time Augmentation approach introduced in this study.

## Figures and Tables

**Table 1:** Comparison of Model Architectures and Performance Metrics.

No.	Model Architecture	2 Halves		4 Quadrants		7 Emotions	
		Accuracy	F1 Score	Accuracy	F1 Score	Accuracy	F1 Score
1	Conv1D MaxPooling1D Dropout (0.5) Flatten Dense	74.46%	85.15%	46.9%	25.48%	23.41%	22.08%
2	Conv1D Dropout (0.25) MaxPooling1D Flatten Dense (128, relu) Dropout (0.25) Dense	73.88%	84.00%	49.87%	35.46%	28.76%	29.95%
3	Conv1D Dropout (0.5) MaxPooling1D Flatten Dense (128, relu) Dropout (0.5) Dense	73.92%	84.20%	49.48%	32.31%	27.35%	25.88%

**Table 2:** Two-Halves classification accuracy (%) under different data-cleaning thresholds, comparing models with and without PCA (mean only, 5-fold CV).

Cleaning Ratio	Accuracy w/o PCA				Accuracy w PCA (0.99)		
	Original	Avg-Aug	Gaussian-Aug	Time-Aug	Original	Avg-Aug	Time-Aug
None	74.2	74.1	74.34	72.4	74.9	73.7	73.0
0	75.1	74.5	74.73	72.5	75.3	74.2	73.8
0.2	75.6	74.8	75.93	73.0	75.9	75.9	74.2
0.4	76.6	74.9	76.43	74.1	77.5	76.6	73.8
0.6	78.7	75.8	78.00	75.5	78.9	78.2	76.2
0.8	82.6	82.8	82.32	82.2	82.6	82.1	81.9
0.95	82.5	83.1	82.67	82.2	82.5	82.5	82.5

**Table 3:** Four-Quadrant classification accuracy (%) under different data-cleaning thresholds, comparing models with and without PCA (5-fold CV). Results include Original, Averaging-based, Gaussian, and Time-based augmentations.

Cleaning Ratio	Accuracy w/o PCA				Accuracy w PCA (0.99)		
	Original	Avg-Aug	Gaussian-Aug	Time-Aug	Original	Avg-Aug	Time-Aug
None	49.0	51.7	50.68	50.2	49.8	52.1	50.6
0	50.1	52.3	51.42	50.6	50.8	52.9	51.3
0.2	50.5	53.3	51.69	50.0	51.1	53.8	50.9
0.4	53.0	53.3	53.12	52.8	53.6	54.1	53.2
0.6	52.7	54.9	52.79	54.8	53.5	55.4	55.0
0.8	60.4	60.3	61.35	58.0	60.7	61.1	59.3
0.95	66.5	65.2	64.31	65.2	66.8	65.7	65.8

**Table 4:** Seven-Emotion classification accuracy (%) under different data-cleaning thresholds for Original, Average Augmentation, Gaussian, and Time-Augmentation methods (without PCA), and for Original, Average Augmentation, and Time-Augmentation (with PCA, 0.99 variance ratio). The MAET baseline [30] is included for reference, using the EEG-only configuration.

Cleaning Ratio	Accuracy w/o PCA					Accuracy w PCA (0.99)		
	Original	Avg-Aug	Gaussian	Time-Aug	MAET	Original	Avg-Aug	Time-Aug
None	27.8	32.5	30.1	30.7	26.4	28.1	32.8	30.9
0	28.7	34.3	31.43	31.5	26.70	29.3	34.6	31.9
0.2	29.8	33.8	32.15	32.0	26.93	30.4	34.0	32.5
0.4	31.4	33.2	33.00	33.2	27.48	31.8	33.6	33.5
0.6	29.7	36.0	33.75	32.3	30.10	30.1	36.3	32.8
0.8	36.7	38.8	36.05	41.1	39.28	37.0	39.2	41.3
0.95	44.0	45.4	42.54	45.0	45.94	44.3	45.7	45.4

**Table 5:** Effect of cleaning threshold on the number of retained samples and class distribution.

Cleaning Ratio	Dataset Size	Class 0 Size	Class 1 Size	% of Total Data
0.00	1592	393	1199	99.50
0.20	1545	371	1174	96.56
0.40	1403	325	1078	87.69
0.60	896	191	705	56.00
0.80	325	57	268	20.31
0.95	200	35	165	12.50

**Table 6:** Mean ± SD of accuracy and F1 scores across five folds for the Two-Halves configuration (with and without PCA).

Cleaning Ratio	Original		Avg Aug		Time Aug	
	Accuracy	F1	Accuracy	F1	Accuracy	F1
*Without PCA*						
0	75.1 ± 2.6	85.5 ± 1.6	74.5 ± 2.2	83.1 ± 1.5	72.5 ± 2.0	82.3 ± 1.4
0.2	75.6 ± 2.5	85.8 ± 1.5	74.8 ± 2.0	84.3 ± 1.4	73.0 ± 2.2	83.0 ± 1.5
0.4	76.6 ± 2.8	86.5 ± 1.5	74.9 ± 1.9	84.4 ± 1.2	74.1 ± 1.7	84.0 ± 1.2
0.6	78.7 ± 2.2	88.0 ± 1.3	75.8 ± 2.5	85.4 ± 1.5	75.5 ± 2.3	85.1 ± 1.5
0.8	82.6 ± 3.6	90.4 ± 1.5	82.8 ± 4.0	90.2 ± 2.4	82.2 ± 4.3	89.7 ± 2.6
0.95	82.5 ± 3.5	90.1 ± 1.7	83.1 ± 3.5	90.3 ± 2.2	82.2 ± 3.9	89.9 ± 2.4
*With PCA*						
0	75.3 ± 2.4	85.3 ± 1.6	74.2 ± 2.2	84.0 ± 1.6	73.8 ± 1.5	83.1 ± 1.3
0.2	75.9 ± 2.3	85.8 ± 1.5	75.9 ± 2.7	85.3 ± 2.0	74.2 ± 1.0	84.2 ± 0.9
0.4	77.5 ± 2.4	86.8 ± 1.6	76.6 ± 2.5	85.8 ± 1.8	73.8 ± 1.7	83.9 ± 1.3
0.6	78.9 ± 2.1	88.0 ± 1.3	78.2 ± 1.5	87.3 ± 1.0	76.2 ± 2.0	85.7 ± 1.3
0.8	82.6 ± 3.6	90.4 ± 2.1	82.1 ± 4.4	89.9 ± 2.6	81.9 ± 3.5	89.7 ± 2.2
0.95	82.5 ± 2.9	90.4 ± 1.7	82.5 ± 2.9	90.4 ± 1.7	82.5 ± 2.9	90.4 ± 1.7

**Table 7:** Summary of Best F1 Scores Across Emotion Classification Setups.

Setup	Method	Threshold	Best F1 (%)	Original F1 (%)	Improvement
2 Halves	Any Aug w PCA	0.8–0.95	90.4	84.33	+7.2%
4 Quadrants	Avg Aug	0.2	44.1	34.0	+29.7%
7 Emotions	Avg Aug	0.95	38.6	27.8	+38.8%

*Note*: Threshold denotes the data-cleaning ratio used to remove low-quality samples. Higher thresholds correspond to stricter noise filtering. F1 values represent mean performance over five-fold cross-validation.

## Data Availability

The data presented in this study are openly available in [SEED Dataset] at [https://bcmi.sjtu.edu.cn/~seed/seed-vii.html].
